# Gut-ovary axis in polycystic ovary syndrome: mechanistic insights and gut microbiota-targeted therapeutic strategies

**DOI:** 10.3389/fendo.2025.1684492

**Published:** 2025-11-05

**Authors:** Mei Zhao, Danlin Chen, Xiumei Hu, Caiping Xie, Lianwei Xu, Fuhua Zhou

**Affiliations:** ^1^ Department of Gynecology, Longhua Hospital, Shanghai University of Traditional Chinese Medicine, Shanghai, China; ^2^ Department of Gynecology, Sanming Hospital of Integrated Medicine, Sanming, China

**Keywords:** polycystic ovary syndrome, gut-ovary axis, gut microbiota, metabolic disorders, novel treatment

## Abstract

Polycystic ovary syndrome (PCOS) is a common endocrine and metabolic disorder that significantly affects women’s reproductive health and quality of life. Its pathogenesis involves multiple factors, including genetics, environment, and metabolism. In recent years, with the growing body of research on PCOS, the “gut-ovary axis” hypothesis has become a prominent research focus. This hypothesis suggests that an imbalance in gut bacteria may significantly influence the onset and progression of PCOS through various pathways, such as immune regulation, metabolic disturbances, and hormonal imbalances. This article aims to review the role of the “gut-ovary axis” in PCOS and to explore novel treatment strategies based on gut microbiota modulation, including probiotics, fecal microbiota transplantation, and dietary interventions. These strategies represent promising research avenues for future PCOS treatments, with preliminary studies demonstrating their potential to improve clinical symptoms. However, it is crucial to note that these are not yet established therapies and require substantial further validation. Novelty and Significance of this Review: This review moves beyond a descriptive catalog of associations to provide a critical appraisal of the gut-ovary axis in PCOS. We systematically differentiate well-established mechanisms from speculative hypotheses, explicitly identify persistent knowledge gaps, and evaluate the translational potential of microbiota-targeted therapies, thereby offering a refined framework for future basic and clinical research.

## 1 Introduction

Polycystic ovary syndrome (PCOS) is a common endocrine disorder primarily affecting women of reproductive age, marked by high androgen levels, ovulation issues, and polycystic changes in the ovaries. Traditionally, research has focused on abnormalities in the hypothalamic-pituitary-ovarian (HPO) axis, but in recent years, studies on the “gut-ovary axis” hypothesis have gained attention. This axis conceptualizes a complex, bidirectional communication network between the gut microbiota and ovarian physiology. It is important to note that this interaction is not direct; rather, it is mediated through integrated intermediary pathways including neuroendocrine signaling (the gut-brain-ovary pathway), bile acid metabolism, and immunometabolic regulation, which collectively influence PCOS pathogenesis.

Recent studies have shown that the diversity of gut microbiota in people with PCOS is reduced, with changes in the abundance of specific bacterial groups, which are associated with metabolic and reproductive disorders in PCOS (1). For example, the hyperinsulinemia commonly seen in people with PCOS may be related to dysbiosis of the gut microbiota, as certain gut bacteria can influence clinical manifestations of PCOS by regulating host energy metabolism and hormone secretion (2, 3).

Moreover, research has found that short-chain fatty acids (SCFAs) produced by gut bacteria play a crucial role in regulating metabolism and inflammatory responses. These metabolites not only affect insulin sensitivity but also may exacerbate PCOS symptoms by influencing endocrine function (4, 5). One key mechanism involves the production of short-chain fatty acids (SCFAs) by gut bacteria, which, for instance, include butyrate—an important SCFA that can promote ovarian health by regulating inflammation and improving intestinal barrier function.

In terms of treatment, treatments aimed at gut bacteria, such as probiotics and dietary adjustments, have shown potential in improving PCOS symptoms. Studies indicate that specific probiotics can promote the restoration of gut microbiota, improve metabolic status, and significantly enhance ovarian function and hormone levels in animal models (6, 7). Additionally, the application of traditional Chinese medicine and other natural products has been found to assist in the treatment of PCOS by modulating gut microbiota, providing new directions for future treatment strategies (8, 9).

In summary, a growing body of evidence suggests that the gut-ovary axis may play an important role in the pathogenesis of PCOS, although the precise mechanisms and causal relationships are still being unraveled. A deeper understanding of the changes in gut microbiota and their impact on ovarian function will aid in the development of novel treatment strategies, providing a more effective theoretical basis for clinical management of PCOS. Future research needs to further explore the complex interactions between gut microbiota and PCOS to offer more personalized and effective treatment options for people with PCOS.

## 2 Main body

### 2.1 Epidemiological association between PCOS and gut microbiota dysbiosis

#### 2.1.1 Characteristics of gut microbiota composition in PCOS patients

Recent studies have shown a significant association between polycystic ovary syndrome (PCOS) and gut microbiota dysbiosis. Specifically, the diversity of gut microbiota in patients with PCOS is significantly reduced, accompanied by abnormal changes in the proportions of Firmicutes and Bacteroidetes, which can affect the patients’ metabolic and hormonal balance (10, 11). The observations indicate that changes in the abundance of specific genera are closely related to the severity of PCOS symptoms. For example, the decrease in the abundance of Prevotella and Bifidobacterium is thought to be associated with the worsening of symptoms and metabolic abnormalities in patients with PCOS (12, 13). Moreover, systematic reviews have shown that the gut microbiota of patients with PCOS exhibits enrichment of genera such as Bacteroides, Enterococcus, and Escherichia-Shigella across multiple studies, indicating that changes in gut microbiota are directly related to the pathological processes of PCOS, independent of obesity (14, 15).

Further research has shown that the characteristics of gut microbiota composition in patients with PCOS are closely related to their clinical manifestations. Specifically, changes in gut microbiota may lead to abnormalities in bile acid and lipid metabolism. These abnormalities exacerbate insulin resistance and low-grade inflammation, which are typical features of PCOS (13, 16). Additionally, some studies emphasize the relationship between gut microbiota and the pathogenesis of PCOS. They suggest that changes in gut microbiota can affect the secretion of sex hormones, thereby influencing ovarian function (17, 18).

In conclusion, in-depth research on the characteristics of gut microbiota in patients with PCOS can provide new ideas for future treatment strategies, especially considering the possibility of improving PCOS-related symptoms by altering gut microbiota. This supports the use of probiotics or other microbial interventions (19, 20).

#### 2.1.2 Relationship between dysbiosis of gut microbiota and the metabolic phenotype of PCOS

Dysbiosis of gut microbiota plays a crucial role in the development of the metabolic phenotype in polycystic ovary syndrome (PCOS). Studies indicate that dysbiosis of gut microbiota may exacerbate insulin resistance in PCOS patients by affecting bile acid metabolism and insulin sensitivity (10, 12). Specifically, the release of endotoxins (such as lipopolysaccharides, LPS), resulting from changes in gut microbiota is considered a key factor in triggering low-grade chronic inflammation. This inflammation can further promote fat accumulation and hormonal imbalances, thereby worsening metabolic abnormalities in PCOS (13, 18).

In several studies, comparisons between PCOS patients and healthy controls have shown that the gut microbiota of PCOS patients exhibits enrichment of specific metabolic pathways. These pathways are particularly related to glucose and lipid metabolism (15, 20). Changes in these metabolic pathways contribute to the patients’ insulin resistance, obesity, and other features of metabolic syndrome, suggesting that dysbiosis of gut microbiota may be an important pathogenic factor in the metabolic phenotype of PCOS.

Moreover, research has found that interventions aimed at improving gut microbiota (such as probiotics and dietary adjustments) can significantly enhance the metabolic status of PCOS patients, reduce insulin resistance, and promote metabolic health, demonstrating the potential therapeutic value of gut microbiota in managing PCOS (12, 21).

Thus, the relationship between dysbiosis of gut microbiota and the metabolic phenotype of PCOS indicates that interventions targeting gut microbiota may provide new treatment strategies for PCOS patients. These strategies could particularly improve metabolic abnormalities and enhance quality of life (10, 17) (**Table 1**).

**Table 1:** Summary of gut microbiota alterations, metabolic consequences, and clinical correlates in PCOS.

Category	Specific changes	Explanation
Changes in Gut Microbiota	↓ Overall microbial diversity ↑ Abundance of Bacteroides/Escherichia-Shigella ↓ Abundance of Prevotella/Bifidobacterium/Akkermansia	Reduced overall microbial diversity and compositional shifts in specific bacterial taxa.
Changes in Microbiota-Derived Metabolites	↓ Short-Chain Fatty Acids (SCFAs), particularly Butyrate ↑ Lipopolysaccharides (LPS) Altered Bile Acid Profiles	Decreased levels of beneficial anti-inflammatory metabolites (SCFAs) and increased levels of pro-inflammatory molecules (LPS).
Associated GI Symptoms	Increased prevalence of bloating, visceral pain, and Irritable Bowel Syndrome (IBS) Potential association with increased intestinal permeability (“leaky gut”)	GI symptoms may arise from dysbiosis-driven inflammation, abnormal gas production, and dysfunction of the gut-brain axis.
Hormonal & Clinical Alterations in PCOS	Metabolic: Insulin Resistance, Obesity Reproductive: Hyperandrogenism, Menstrual Irregularity, Anovulation Inflammatory: Chronic Low-Grade Inflammation	Microbial and metabolic changes contribute directly and indirectly to the core clinical features of PCOS.

"↑" indicates an increase or up-regulation.

"↓" indicates a decrease or down-regulation.

#### 2.1.3 The role of dietary habits and gastrointestinal symptoms

Dietary habits constitute a primary environmental factor shaping the gut microbiota ecosystem. Many women with PCOS report dietary patterns high in saturated fats and refined carbohydrates but low in dietary fiber. This “Western-style” diet is known to reduce the abundance of beneficial, SCFA-producing bacteria (e.g., Bifidobacterium, Akkermansia) while promoting the expansion of pro-inflammatory microbial pathobionts. Consequently, such a diet can exacerbate intestinal barrier dysfunction, increase systemic LPS levels (metabolic endotoxemia), and fuel chronic low-grade inflammation, thereby aggravating both metabolic and reproductive aspects of PCOS.

Clinically, the disturbances of the gut-ovary axis often manifest as functional gastrointestinal symptoms. A growing body of evidence suggests a high co-morbidity between PCOS and disorders like Irritable Bowel Syndrome (IBS), with patients frequently reporting bloating, abdominal discomfort, and visceral pain. These symptoms are not merely coincidental but may be direct consequences of the underlying dysbiosis, altered gut motility, and heightened visceral sensitivity mediated by the gut-brain axis. Recognizing and assessing these GI symptoms in PCOS patients is crucial, as they represent a tangible clinical manifestation of gut dysbiosis and may be a target for dietary and probiotic interventions aimed at improving overall well-being beyond core PCOS symptoms.

### 2.2 Molecular mechanisms of the “gut-ovary axis”

#### 2.2.1 Regulatory role of short-chain fatty acids

Short-chain fatty acids (SCFAs), such as butyrate, propionate, and acetate, are primarily produced by gut microbiota through the fermentation of dietary fibers and have significant effects on the host’s metabolism and immune function (22). A body of evidence primarily from preclinical models indicates that SCFAs may improve insulin sensitivity by activating G protein-coupled receptors (GPR41 and GPR43) (22). However, direct confirmation of this specific signaling pathway as the primary mechanism in humans with PCOS is still lacking. This mechanism is particularly relevant for patients with PCOS, as improved insulin sensitivity can alleviate some of the metabolic disturbances associated with the condition. *In vitro* studies and rodent models suggest that SCFAs like butyrate may inhibit ovarian androgen synthesis (23, 24). This represents a compelling direct mechanism for the gut-ovary axis; however, it remains to be determined whether physiological concentrations of SCFAs from gut fermentation can reach the human ovary to exert this effect directly, or if the benefits are primarily mediated through systemic improvement in metabolism and inflammation. A critical comparison across studies, however, reveals that the most direct evidence for this inhibition comes from *in vitro* models of ovarian theca cells and pre-clinical rodent models of PCOS (23). The translation of these findings to humans requires further validation, as human data are often correlational. Mechanistically, beyond receptor activation, SCFAs are also known inhibitors of histone deacetylases (HDACs). This epigenetic regulation represents another potent pathway through which they could modulate the expression of genes involved in steroidogenesis, such as CYP17A1. Nevertheless, the precise molecular cascade from GPR activation or HDAC inhibition to the transcriptional regulation of androgen-producing enzymes in the human ovary remains an active and critical area of investigation. Furthermore, SCFAs influence systemic inflammation and metabolic status by modulating gut microbiota composition, which plays a crucial role in the pathogenesis of PCOS (9, 25). Beyond SCFAs, other gut microbiota-derived metabolites, such as indole derivatives and polyamines, have also been implicated in the pathophysiology of PCOS, as comprehensively discussed in a recent review (26).Research shows that SCFAs are important in regulating energy metabolism, reducing insulin resistance, and promoting the recovery of ovarian function. Thus, interventions targeting SCFAs may offer novel therapeutic strategies for the treatment of PCOS.

#### 2.2.2 Involvement of bile acid metabolism pathways

Bile acids are bioactive molecules synthesized by the liver that are involved in the digestion and absorption of fats. Secondary bile acids (such as deoxycholic acid) are biotransformed by gut microbiota in the intestine, which regulate the hepatic glucose and lipid metabolism by binding to the farnesoid X receptor (FXR) in the liver. Studies indicate that bile acid metabolism not only affects liver metabolic function but may also indirectly influence the occurrence and development of PCOS by affecting ovarian function (9, 27). Bile acids play a key role in regulating insulin sensitivity, cholesterol metabolism, and energy balance. In PCOS patients, abnormal bile acid metabolism is closely related to insulin resistance and inflammatory responses (28, 29). A key question is how systemic bile acid signaling intersects directly with ovarian pathology. The farnesoid X receptor (FXR) and Takeda G protein-coupled receptor 5 (TGR5) are expressed in ovarian tissue. Their activation creates a direct molecular link to steroidogenesis. FXR activation, both systemically (via FGF19) and locally within the ovary, can suppress the expression of key steroidogenic enzymes. Conversely, TGR5 signaling through cAMP may have a more complex, context-dependent role, as cAMP is a classic stimulator of steroidogenesis. This duality suggests that the net effect of bile acid signaling on ovarian androgen production depends on the specific receptor activated, the local bile acid composition, and the metabolic context, representing a nuanced layer of regulation in PCOS. Therefore, studying the metabolic pathways of bile acids and their interactions with gut microbiota will provide new directions for the prevention and treatment of PCOS.

#### 2.2.3 Gut-brain-ovary neuroendocrine pathway

**Figure 1:**
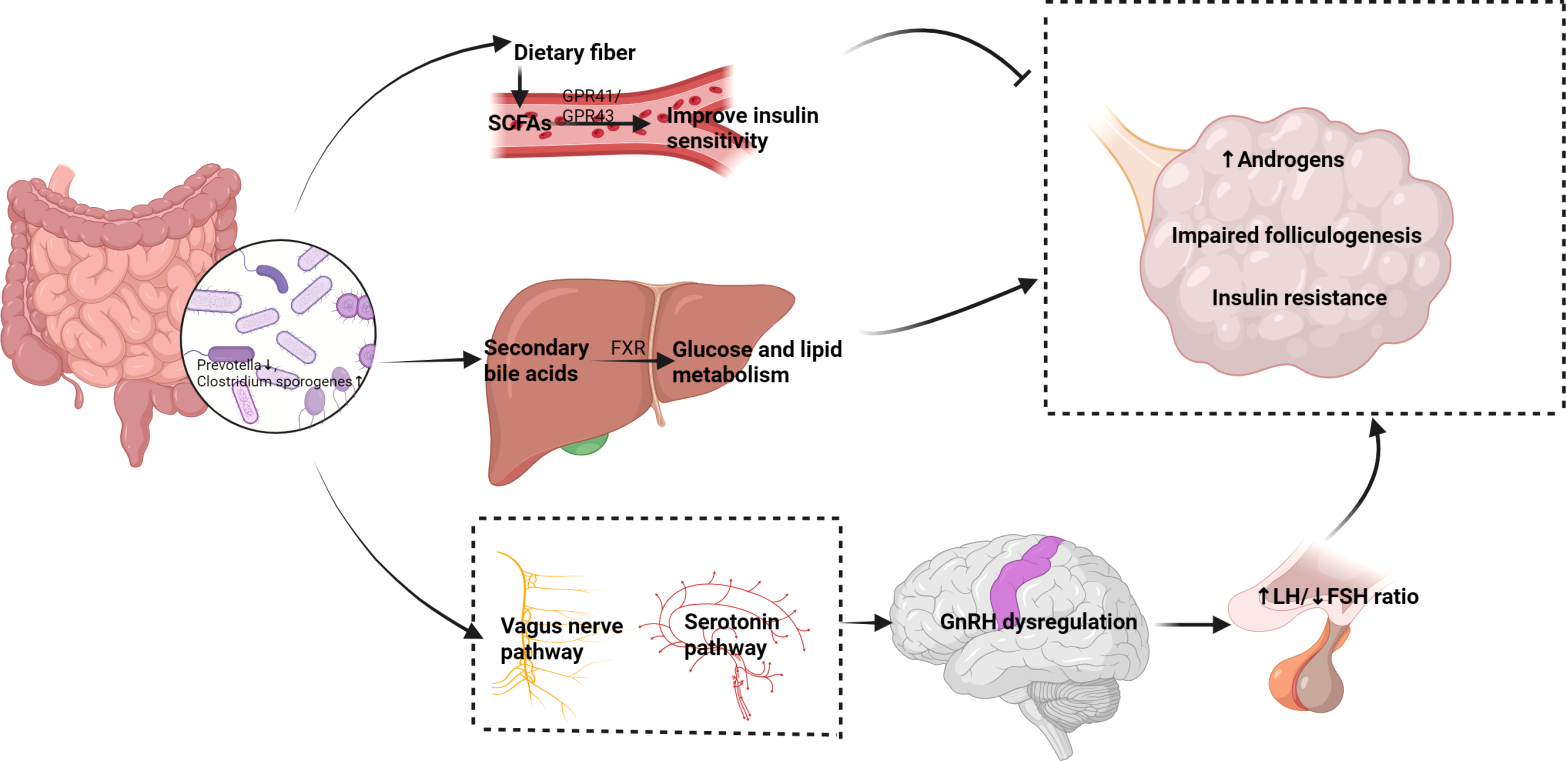
Proposed mechanisms of the gut-ovary axis in PCOS pathogenesis. Dysbiosis of gut microbiota (left) affects PCOS through multiple pathways (1): SCFAs (e.g., butyrate) improve insulin sensitivity and inhibit ovarian androgen synthesis via GPR41/43 (2); Secondary bile acids regulate glucose/lipid metabolism through FXR (3);The gut-brain-ovary neuroendocrine pathway disrupts GnRH secretion. These interactions collectively contribute to hyperandrogenism, insulin resistance, and follicular dysfunction (right).

The interaction between the gut and the central nervous system is referred to as the gut-brain axis, and recent studies have found that this pathway is also important in regulating reproductive function. Gut microbiota can influence the activity of gonadotropin-releasing hormone (GnRH) neurons through the vagus nerve or serotonin pathways, thereby modulating the function of the hypothalamic-pituitary-ovarian (HPO) axis. Research shows that gut microbiota can affect ovarian function by modulating neuroendocrine signals and subsequently influence menstrual cycles and ovulation (2, 30). This gut-brain-ovary neuroendocrine pathway suggests that changes in gut microbiota may influence the synthesis and secretion of ovarian hormones by affecting GnRH release, providing a new perspective on the pathogenesis of PCOS and related reproductive endocrine disorders. Therefore, interventions targeting gut microbiota may become effective strategies for regulating this pathway and improving PCOS symptoms (**Figure 1**).

### 2.3 Impact of gut microbiota dysbiosis on core pathology of PCOS

#### 2.3.1 Mechanism promoting hyperandrogenism

Certain gut bacteria, such as Clostridium sporogenes, play significant roles in polycystic ovary syndrome (PCOS) patients. Studies indicate that these bacteria can inhibit intestinal aromatase activity, leading to elevated testosterone levels. Hyperandrogenism is one of the core features of PCOS, typically accompanied by various metabolic and reproductive dysfunctions. Dysbiosis of the gut microbiota may exacerbate this pathological state by promoting androgen production. Research has found a correlation between changes in gut microbiota composition and elevated serum testosterone levels in PCOS model mice. This indicates that gut microbiota plays an important role in the pathogenesis of PCOS (31). Further analysis shows that changes in gut microbiota metabolites may regulate androgen production by affecting the host’s endocrine environment, leading to hyperandrogenism.

Additionally, metabolites of gut microbiota, particularly short-chain fatty acids (SCFAs), play a crucial role in regulating endocrine balance. SCFAs not only promote energy metabolism but also inhibit inflammation-related cytokines; this helps alleviate insulin resistance and low-grade inflammation, which are common pathological states in PCOS patients (32). Therefore, regulating gut microbiota, especially through dietary interventions, probiotics, and SCFA supplementation, may become an important strategy for reducing hyperandrogenism in PCOS patients.

#### 2.3.2 Association with follicular development disorders

Dysbiosis of gut microbiota not only affects androgen levels but may also lead to follicular development disorders. Studies indicate that metabolites of gut microbiota, such as indole, can interfere with the function of granulosa cells by activating the aryl hydrocarbon receptor (AhR). This interference subsequently inhibits the maturation of follicles. Granulosa cells play a key role in follicular development, and their dysfunction directly impacts follicle growth and development (1). In patients with Polycystic Ovary Syndrome (PCOS), alterations in gut microbiota composition and metabolic function are closely related to follicular development disorders, further exacerbating reproductive issues.

Building upon these findings, related studies further demonstrate that certain specific compositions of gut microbiota can influence follicle maturation and ovulation by regulating ovarian endocrine function. In PCOS models, reduced diversity of gut microbiota and changes in the abundance of specific bacterial groups negatively correlate with follicular development disorders (30). Additionally, bacteria that promote follicle maturation, such as Akkermansia, are significantly reduced in PCOS patients. Therefore, regulating gut microbiota and restoring its normal function may be an effective approach to improving follicular development disorders in PCOS.

Supporting this, a recent groundbreaking study identified a specific strain of Lactobacillus reuteri that could ameliorate hyperandrogenism and restore ovarian function in a PCOS mouse model by modulating gut microbiota and improving intestinal barrier integrity, providing a compelling case for targeted bacteriotherapy (33).

In summary, dysbiosis of gut microbiota not only contributes to the occurrence of PCOS by increasing androgen levels but also exacerbates this pathological state by affecting follicular development and granulosa cell function. This finding provides new insights for the treatment of PCOS, emphasizing the potential value of gut microbiota in PCOS therapy.

3The mechanistic insights discussed above are predominantly derived from animal studies. While these models are indispensable for uncovering causal relationships within the gut-ovary axis, it is crucial to acknowledge their inherent limitations. Commonly used PCOS models (e.g., induced by letrozole, testosterone, or high-fat diet) capture specific facets of the syndrome but fail to mimic its full spectrum of heterogeneity seen in patients. Furthermore, significant interspecies differences in anatomy, gut microbiota ecology, and immune function mean that therapeutic strategies showing efficacy in rodents may not directly translate to humans. Therefore, the promising findings from pre-clinical research must be interpreted as a guide for hypothesis generation rather than a guarantee of clinical success.

### 2.4 Treatment strategies for PCOS based on gut microbiota modulation

#### 2.4.1 Application of probiotics and prebiotics

It is important to state at the outset that probiotics are not currently a standard or recommended treatment for PCOS in clinical guidelines. However, they show interesting potential in research settings. Studies indicate that supplementation with the genera Lactobacillus and Bifidobacterium can significantly reduce insulin resistance and reduce elevated testosterone levels. Clinical trial results show that after supplementation with these probiotics, insulin sensitivity in PCOS patients was effectively enhanced, and symptoms caused by elevated testosterone levels were alleviated (34). Probiotics regulate the composition of gut microbiota and reduce intestinal inflammation. These effects subsequently influence systemic metabolism and hormone levels. This is particularly important in PCOS patients, as this condition is often accompanied by metabolic disorders and endocrine imbalances. A critical appraisal of the human trials in this field, however, reveals significant challenges. A major source of inconsistency is the profound heterogeneity in study design. First, there is a wide variation in the probiotic strains and combinations used across different trials, and the effects are often strain-specific. Second, the choice of primary and secondary outcome measures varies considerably, ranging from biochemical markers (HOMA-IR, testosterone) to clinical features (menstrual regularity, acne), making direct comparison and meta-analysis difficult. Furthermore, many studies are limited by small sample sizes, short follow-up periods, and a lack of parallel dietary control, which may confound the results. Consequently, while the existing data are promising, they fall short of providing definitive, generalizable guidelines for probiotic use in PCOS. Future large-scale, well-designed, randomized controlled trials employing standardized outcomes and specific, well-defined microbial strains are urgently needed.

Moreover, prebiotics, as “food” for probiotics, are also crucial for gut health. By promoting the growth of beneficial bacteria, prebiotics can indirectly improve insulin sensitivity and reduce inflammatory responses, thereby playing a role in the management of PCOS (1).A valid safety consideration is whether prebiotics could also promote the growth of pathogenic bacteria. However, established prebiotics are defined by their selective utilization, meaning they are not efficiently metabolized by most harmful species. Clinical studies in PCOS and related metabolic conditions have not reported enrichment of pathogenic microbiota with prebiotic intervention; instead, a shift towards a healthier microbial community is typically observed (35, 36). The most common adverse effects, such as mild and transient bloating, are generally manageable and underscore the importance of dose titration.

Despite the positive results of probiotics and prebiotics in the treatment of PCOS, current research still needs to further validate their long-term effects and safety, especially regarding their applicability and effectiveness in different populations. Future research could consider exploring specific strains and their metabolites as therapeutic targets to provide a more solid foundation for personalized treatment of PCOS (30, 37).

#### 2.4.2 Potential of fecal microbiota transplantation

Fecal microbiota transplantation (FMT) is a therapeutic strategy with established efficacy in recurrent Clostridioides difficile infection and is now being explored for a range of other dysbiosis-associated conditions. In the context of PCOS, it represents an emerging treatment strategy that shows potential in restoring ovarian function and improving PCOS symptoms in preliminary research. Animal studies indicate that FMT can restore normal ovarian cycles in a PCOS mouse model and improve the endocrine function and metabolic abnormalities of these mice. These research results lay the foundation for the application of FMT in the treatment of PCOS, although human studies are still in exploratory stages and require verification of its safety and effectiveness (38).

Mechanistically, FMT aims to improve gut dysbiosis by reconstructing microbial communities, thereby influencing hormone levels and metabolic status. Two common questions regarding this process are the necessity of pre-conditioning and the fate of transplanted microbes. Contrary to intuition, complete eradication of the recipient’s native microbiota via antibiotics is not a prerequisite for FMT. The procedure’s efficacy often relies on the competition and synergy between the transplanted donor microbiota and the resident community. Furthermore, the engraftment of donor bacteria is a dynamic and partial process. Not all transplanted species permanently colonize the gut; however, even transient shifts in the microbial landscape and its metabolic functions can be sufficient to disrupt pathological states and initiate therapeutic recovery. The long-term success of FMT likely depends on stabilizing these beneficial changes through sustained dietary and lifestyle factors. Recent studies have found that FMT not only restores the diversity of gut microbiota but is also associated with improvements in PCOS-associated symptoms such as insulin resistance and hyperandrogenism (39). These findings suggest that FMT may become an effective treatment for PCOS patients; however, large-scale clinical trials to assess its safety and long-term effects are essential.

**Figure 2:**
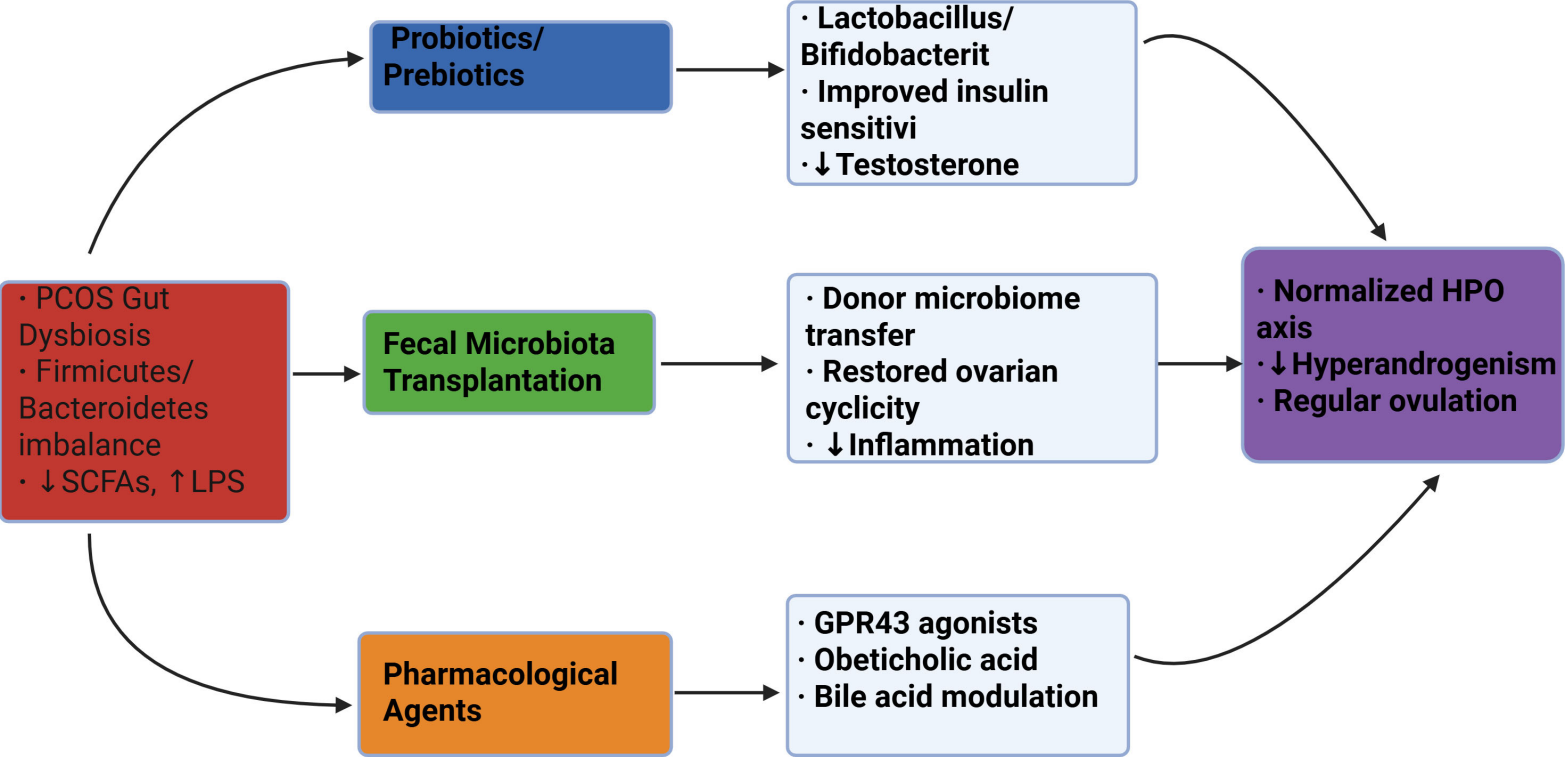
Schematic of microbiota-targeted therapies for PCOS. Three primary intervention approaches (color-coded) address gut dysbiosis through distinct mechanisms, ultimately improving clinical outcomes. SCFAs, short-chain fatty acids; LPS, lipopolysaccharide; HPO, hypothalamic-pituitary-ovarian.

In summary, while the clinical application of FMT for PCOS is still in its infancy and requires validation through large-scale controlled trials, the compelling evidence from preclinical models and its success in other fields position it as a strategy with significant potential. Future work will need to focus on standardizing protocols and ensuring long-term safety (**Figure 2**).

Knowledge Gap and Critical Perspective: Despite promising animal data, the clinical application of FMT for PCOS faces significant hurdles. Key unknowns include the long-term stability of the transplanted microbiota, the optimal donor profile, and the potential for serious adverse events. Furthermore, the mechanistic basis for its efficacy—whether it requires permanent engraftment of specific taxa or merely a transient “reset” of the microbial ecosystem—is purely speculative and represents a critical area for future research.

### 2.5 Development of novel drugs targeting the “gut-ovary axis”

#### 2.5.1 Preclinical studies of SCFA receptor agonists (e.g., GPR43 agonists)

Short-chain fatty acids (SCFAs) play an important role in the metabolic processes mediated by gut microbiota, particularly through their action in activating G protein-coupled receptors (GPRs), influencing various physiological and pathological processes. Studies indicate that SCFAs may play an important role in the pathogenesis of polycystic ovary syndrome (PCOS) by regulating metabolism, immune responses, and inflammation. GPR43 agonists represent a new direction for drug development and have shown potential therapeutic effects in preclinical studies. According to existing literature, SCFAs can improve the metabolic status and ovarian function of PCOS patients by promoting insulin sensitivity and reducing inflammation (18).

In preclinical trials, GPR43 agonists have shown regulatory effects on adipocytes, reducing insulin resistance and lowering blood glucose levels. This is particularly important for PCOS patients, as this condition is often accompanied by insulin resistance. By modulating the composition and activity of gut microbiota, GPR43 agonists may indirectly influence ovarian function, which in turn could lead to improved fertility. Additionally, the application of GPR43 agonists in animal models has shown effective reduction of inflammatory markers systemically, further supporting their potential in the treatment of PCOS.

However, despite encouraging preliminary results, more preclinical studies are needed to determine the specific mechanisms of action of GPR43 agonists in PCOS patients. Additionally, their safety and effectiveness require thorough evaluation. Future research should focus on the dose-dependent effects of this drug, long-term safety, and the combined effects with existing treatment regimens.

#### 2.5.2 Progress of bile acid analogs (e.g., obeticholic acid) in patients with PCOS and NAFLD

Bile acids play an important role in regulating metabolism and maintaining the stability and diversity of gut microbiota. In recent years, studies have found that bile acid analogs such as obeticholic acid may have beneficial therapeutic effects on patients with polycystic ovary syndrome (PCOS) and concomitant non-alcoholic fatty liver disease (NAFLD). As a selective farnesoid X receptor (FXR) agonist, obeticholic acid has shown good prospects in the treatment of diabetes and liver diseases.

In patients with PCOS and NAFLD, abnormal bile acid levels are closely related to metabolic disorders. Studies indicate that obeticholic acid can improve the metabolic status of PCOS patients by enhancing liver lipid metabolism and reducing liver inflammatory responses. Additionally, it can improve liver function by increasing insulin sensitivity (18). Moreover, obeticholic acid may promote metabolic health and alleviate PCOS symptoms by modulating the composition of gut microbiota.

Currently, clinical trials on obeticholic acid in patients with PCOS and NAFLD are being conducted, with preliminary results showing its potential in improving liver function and metabolic indicators. However, further randomized controlled trials and large-scale clinical studies are needed to verify its long-term safety and effectiveness. Future research directions should include investigating the impact of different doses and treatment durations on patients. In addition, studies on the combined effects of obeticholic acid with other treatment regimens are necessary to provide a more comprehensive strategy for the treatment of PCOS and its comorbidities (**Table 2**).

**Table 2:** Smmmary of clinical evidence for gut microbiota-targeted therapies in PCOS.

Intervention	Study type	Participants/model	Duration	Metabolic outcomes	Reproductive/hormonal outcomes	Inflammatory outcomes	Ref.
Probiotics (Lactobacillus)	Clinical trial	PCOS women (n=60)	12 weeks	↓HOMA-IR, ↓ Fasting Insulin	↓ Total Testosterone, ↑ Menstrual Regularity	↓ Serum CRP	(34)
Fecal Microbiota Transplantation (FMT)	Animal study	Letrozole-induced PCOS mouse model	4 weeks	Improved Glucose Tolerance, ↓ Body Weight Gain	Restored Ovarian Cyclicity, ↓ Serum Testosterone	↓ Ovarian TNF-α, IL-6	(38)
Obeticholic acid	Preclinical trial	PCOS rat model with NAFLD	8 weeks	↓ Liver Triglycerides, Improved Insulin Sensitivity	↓ Ovarian Cyst Number, ↓ Androgen Levels	↓ Hepatic Inflammatory Infiltration	(18)

"↑" indicates an increase or up-regulation.

"↓" indicates a decrease or down-regulation.

### 2.6 Limitations of current research

#### 2.6.1 Insufficient causal evidence of microbiota-host interactions

Current research on polycystic ovary syndrome (PCOS) and its relationship with gut microbiota primarily relies on cross-sectional designs. These designs limit our understanding of the causal relationships between microbiota and the host. While cross-sectional studies can reveal correlations, they cannot provide causal evidence. For example, one study reported a significant association between the composition of gut microbiota and the metabolic health status of PCOS patients. However, due to the lack of longitudinal data, the temporal sequence and biological mechanisms underlying this relationship remain unclear (40). Moreover, gene-edited animal models should be more widely applied, as they can precisely explore the causal relationships between specific microbial populations and host physiology in controlled environments. For instance, experiments by Ridura et al. demonstrated that transplanting human microbiota into mice could reproduce the obesity phenotype of human donors, providing a good model for exploring the specific effects of microbiota on host metabolism and endocrine function (41). Therefore, future research should adopt more longitudinal cohort designs and gene-edited models to comprehensively reveal causal relationships in microbiota-host interactions.

#### 2.6.2 Challenges of personalized treatment

Personalized treatment faces numerous challenges in the management of PCOS, particularly with regard to interventions targeting the gut microbiota. The effectiveness of these approaches is often influenced by various factors such as host genotype, baseline diet, and lifestyle. Research indicates that the genomic characteristics of hosts can significantly affect the composition of their microbiota, leading to different responses to specific treatments (42). For example, some individuals may respond more significantly to dietary fiber than others; this variability is closely related to their baseline gut microbiota composition (43). Therefore, when implementing personalized microbiome intervention strategies, it is essential to consider these variables to ensure the effectiveness of the treatments and reduce variability in treatment outcomes. Moreover, implementing precise host phenotyping combined with microbiome data requires a comprehensive assessment to develop more effective personalized treatment plans. This approach can help achieve more targeted interventions in the management of PCOS, thereby improving people’s overall health (44).

#### 2.6.3 Regulatory and ethical considerations

Beyond the scientific challenges, the translation of gut microbiota-targeted therapies, particularly FMT and live biotherapeutics, faces significant regulatory and ethical hurdles. From a regulatory standpoint, FMT occupies an ambiguous category. While it may be considered a tissue transplant in some contexts, its use for conditions like PCOS is typically regulated as an investigational drug by agencies such as the U.S. Food and Drug Administration (FDA) and the European Medicines Agency (EMA). This requires adherence to stringent Investigational New Drug protocols, including Good Manufacturing Practice for production, which poses a substantial barrier to widespread clinical adoption. Defined consortia of bacteria or single-strain therapies are unequivocally classified as drugs, mandating a rigorous, phased clinical development pathway that is both time-consuming and costly.

The ethical landscape is equally complex. The long-term safety profile of FMT is not yet fully understood, raising questions about potential unintended consequences on metabolism, immune function, or even behavior. This necessitates not only exhaustive donor screening but also a sophisticated process of informed consent, ensuring patients comprehend the potential for unknown long-term risks. Furthermore, the commercialization of human-derived microbial products introduces ethical dilemmas regarding donor compensation, patenting of natural biological entities, and ensuring equitable access to these potentially expensive therapies across different socioeconomic groups. Addressing these regulatory and ethical issues in parallel with scientific advancement is critical for the responsible and successful integration of microbiome-based interventions into PCOS clinical care.

### 2.7 Future research directions

#### 2.7.1 Multi-omics integration analysis

In future research, multi-omics integration analysis will become an important tool for understanding polycystic ovary syndrome (PCOS) and its related mechanisms. Multi-omics integration analysis involves collecting omics data at different levels (such as genomics, transcriptomics, metabolomics, etc.) to provide a more comprehensive understanding of biological mechanisms. By combining metagenomics, metabolomics, and single-cell sequencing technologies, researchers can conduct in-depth analyses of the molecular networks between gut microbiota and ovaries. This approach can not only reveal the complex interactions between microbiota and host biology but can also provide new biomarkers and therapeutic targets for the development and treatment of PCOS. For example, studies have shown that single-cell multi-omics technologies can effectively capture intercellular heterogeneity, helping to identify specific cell populations and their functions related to PCOS (45). Moreover, utilizing advanced data analysis methods such as machine learning and deep learning can more accurately and efficiently integrate and analyze large volumes of omics data, thereby promoting the development of personalized medicine (46, 47). Therefore, future research should focus on constructing and optimizing multi-omics integration analysis frameworks to better understand the complexity of PCOS and to provide more precise treatment strategies.

#### 2.7.2 Optimization of microbial combination therapy

**Figure 3:**
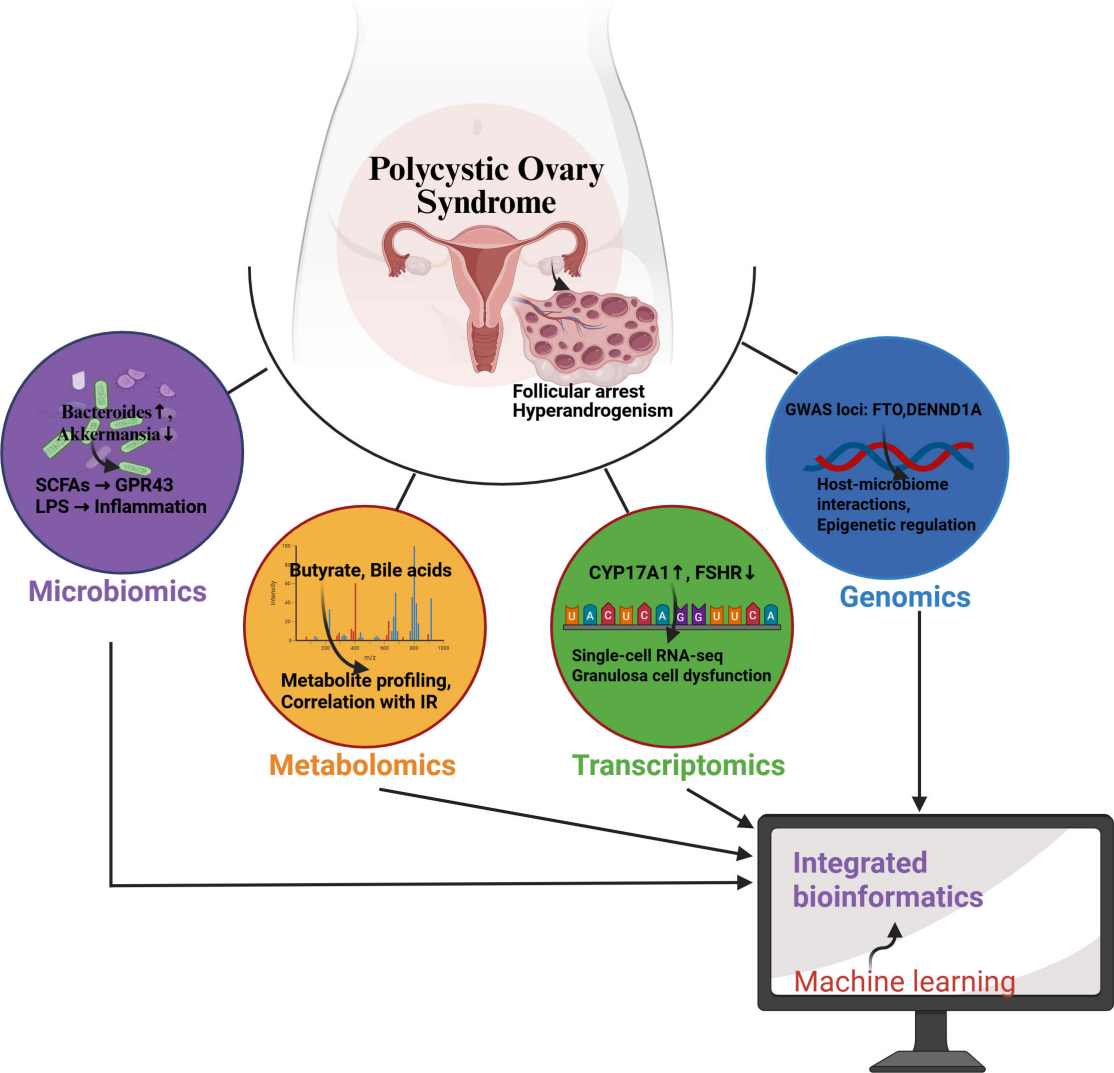
Schematic of multi-omics integration for gut-ovary axis research in PCOS. The network depicts how genomics (blue), transcriptomics (green), metabolomics (orange), and microbiome data (purple) converge on Machine learning ovarian dysfunction in PCOS. Computational integration (gray) identifies key biomarkers and pathways.

Optimizing microbiota-based combination therapy is another important direction for future research in the treatment of PCOS. Studies indicate that the composition of gut microbiota is closely related to the occurrence and development of PCOS (48). Therefore, exploring the synergistic mechanisms of probiotics combined with traditional drugs (such as metformin) will provide new insights for improving the metabolic status and reproductive health of PCOS patients. Specifically, the use of probiotics can improve insulin sensitivity by modulating gut microbiota, thereby combating metabolic disorders associated with PCOS (35, 36). Additionally, researchers need to consider how to design optimal dosing regimens to ensure effective synergy between probiotics and medications. This may involve optimizing dosing times, dosages, and individualized treatment strategies to maximize therapeutic effects. For example, the combined use of probiotics and medications can enhance drug efficacy and reduce side effects by adjusting the structure of microbial communities (49). Future research should focus on how to effectively integrate microbial therapies with existing pharmacological treatments to provide more comprehensive and effective treatment options for PCOS patients (**Figure 3**).

Synthesis of Evidence: In this review, we have endeavored to critically appraise the literature on the gut-ovary axis. The strength of evidence varies significantly across different claims (50). We explicitly distinguish between:

Mechanistic insights derived from *in vitro* and animal models, which are crucial for generating hypotheses but require validation in humans. Clinical associations observed in human cross-sectional studies, which can indicate relationships but not causation.

Interventional evidence from human randomized controlled trials (RCTs), which provide the strongest support for therapeutic efficacy. This graded perspective is essential for accurately representing the current state of the field.

## 3 Conclusion

Polycystic ovary syndrome (PCOS) is a common endocrine disorder that affects a large number of women of reproductive age worldwide, and its complex pathogenesis has long troubled clinical and basic research. In recent years, the idea of the gut-ovary axis has opened up new avenues for research on PCOS, which emphasizes the roles of gut microbiota in metabolism, immunity, and neuroendocrine functions. This discovery not only broadens our understanding of the pathological mechanisms of PCOS but also offers new possibilities for personalized treatment.

The diversity of gut microbiota is closely related to health, and dysbiosis is linked to the occurrence of various metabolic diseases. Research shows that the composition of gut microbiota in women with PCOS is significantly different from that of healthy women; moreover, these changes may lead to the occurrence and development of PCOS by affecting the host’s metabolic and immune responses. By modulating gut microbiota, it is possible to influence the production of specific metabolic compounds, improve insulin resistance, and reduce inflammatory responses, thereby providing new intervention strategies for alleviating PCOS symptoms.

Regarding intervention strategies, methods such as probiotics and fecal microbiota transplantation (FMT) show clinical potential; these methods can restore gut microbiota balance to some extent and improve PCOS-related symptoms. However, these strategies still face challenges in clinical application, including individual differences and concerns about long-term safety. Each person’s gut microbiota characteristic differs, leading to significant variations in the effects of probiotics, which highlights the importance of personalized treatment approaches in clinical studies.

Future research should focus more on the integration of mechanistic exploration and translational applications. On one hand, in-depth studies on the interactions between gut microbiota and PCOS will help reveal the underlying pathophysiological mechanisms of the disease and provide a solid theoretical basis for clinical interventions. On the other hand, with the rapid development of precision medicine, researchers need to translate the results of basic research into clinical applications, promoting the formulation and implementation of precise treatment strategies for PCOS.

Overall, research on the gut-ovary axis provides a compelling new perspective for understanding the complex pathogenesis of PCOS and suggests novel avenues for future treatment exploration. However, the translation of these findings into established clinical practice faces significant hurdles, including the need for causal human evidence, standardized interventions, and long-term safety data. The axis should be viewed as an important piece of the PCOS puzzle, complementary to, rather than a replacement for, established pathophysiological concepts and treatments. However, to ensure the clinical efficacy of these treatments, ongoing research and validation will be essential. By strengthening the integration of research and clinical practice, we hope to achieve new breakthroughs in the management and treatment of PCOS, ultimately improving patients’ quality of life.
